# Incorporation of Anions into Anodic Alumina—A New Track in Cr(VI) Anodizing Substitution?

**DOI:** 10.3390/ma17122938

**Published:** 2024-06-15

**Authors:** Katarzyna Tomczyk, Wojciech J. Stępniowski

**Affiliations:** Institute of Materials Science and Engineering, Faculty of Advanced Technologies and Chemistry, Military University of Technology, 2 Kaliskiego Street, 00908 Warsaw, Poland; katarzyna.tomczyk@wat.edu.pl

**Keywords:** anodizing, aluminum alloys, corrosion, chromates, Cr(VI)-free anodizing, incorporation, corrosion inhibitors, sodium molybdate, anodic aluminum oxide

## Abstract

Aluminum technical alloys are well known for their outstanding mechanical properties, especially after heat treatment. However, quenching and aging, which improve the mechanical properties, by the formation of Cu-rich zones and phases that are coherent with the matrix and block the dislocation motion, cause uneven distribution of the elements in the alloy and consequently make it prone to corrosion. One method providing satisfactory corrosion protection of aluminum alloys is anodizing. On an industrial scale, it is usually carried out in electrolytes containing chromates that were found to be cancerogenic and toxic. Therefore, much effort has been undertaken to find substitutions. Currently, there are many Cr(VI)-free substitutes like tartaric–sulfuric acid anodizing or citric–sulfuric acid anodizing. Despite using such approaches even on the industrial scale, Cr(VI)-based anodizing still seems to be superior; therefore, there is an urge to find more complex but more effective approaches in anodizing. The incorporation of anions into anodic alumina from the electrolytes is a commonly known effect. Researchers used this phenomenon to entrap various other anions and organic compounds into anodic alumina to change their properties. In this review paper, the impact of the incorporation of various corrosion inhibitors into anodic alumina on the corrosion performance of the alloys is discussed. It is shown that Mo compounds are promising, especially when combined with organic acids.

## 1. Introduction

**Figure 1:**
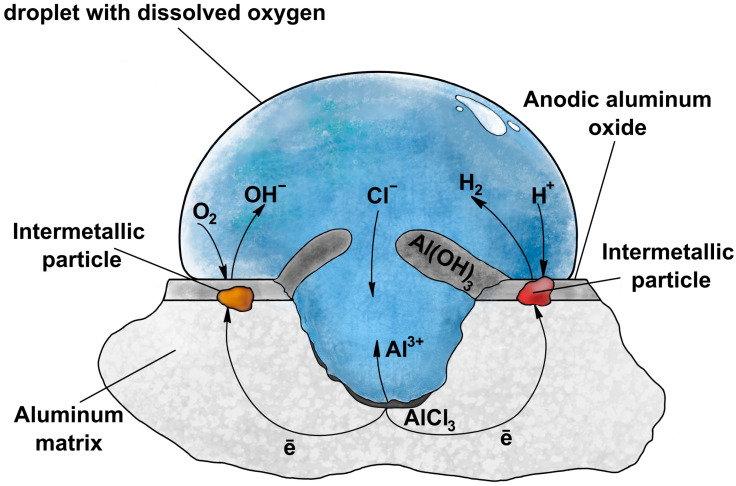
Mechanism of corrosion of aluminum alloy surface.

Heat-treatable aluminum alloys, like 2XXX or 7XXX, have superior mechanical properties accompanied by low density. Thus, these alloys find numerous applications in crucial branches of industry such as aviation, the automotive industry, and electronics. During heat treatment, secondary phases that are coherent with the aluminum matrix are formed and hinder the dislocation motion, which hardens the material. Simultaneously, these secondary, Cu-rich strengthening phases are responsible for poorer corrosion performance when compared to pure, elemental aluminum. Uneven distribution of elements, like the mentioned strengthening phases, results in uneven electrochemical potential distribution on the surface and, consequently, corrosion cells are easily formed, triggering this destructive phenomenon. Thus, Cu-rich regions have greater electrochemical potential and behave like local cathodic sites, where hydrogen reduction or oxygen evolution occurs, while the Cu-poor aluminum matrix behaves like a local anode and corrodes, via oxidation of metallic Al^0^ to Al^3+^ (Figure 1).

**Figure 2:**
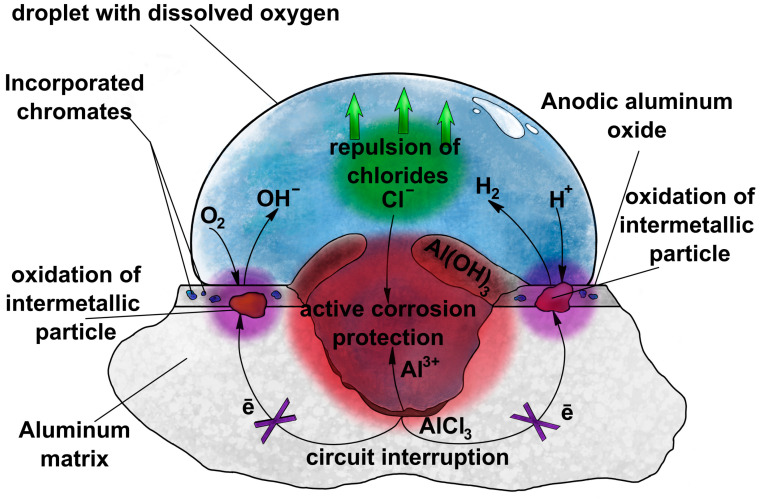
Scheme showing how chromates provide corrosion protection of aluminum alloys.

There are several methods of aluminum alloy corrosion protection, like painting or conversion coatings, but in the long run, they are also vulnerable to corrosion, including filiform and pitting/crevice corrosion. One of the best and most effective corrosion protection approaches is anodizing, i.e., electrochemical oxidation of the aluminum surface in certain electrolytes that provide compact, uniform, and adherent oxide coatings. In industrial practice, three main approaches are commonly used: type I, conducted in chromic acid (CAA—chromic acid anodizing); type II in sulfuric acid (SAA—sulfuric acid anodizing); and type III, conducted in sulfuric acid but at high voltages and usually at lowered temperature, to dissipate Joule’s heat (hard anodizing in sulfuric acid). The best corrosion performance on thin anodic films was found on surfaces after type I anodizing. Cr(VI) anodizing works in a threefold way: incorporated chromates provide active corrosion protection as well as lowering the zeta potential of the oxide and consequently repulse chlorides, hindering corrosion, and simultaneously, chromates in the electrolyte, during anodizing, can oxidize the strengthening intermetallic phases, which breaks the circuit in the corrosion cell [1] (Figure 2). Quantitatively, anodizing in chromates provides high corrosion potential, high pitting potential, and low corrosion current density.

Despite numerous technological advantages, numerous research studies have shown that chromates, even in ppm concentrations, are dangerous. When exposed to chromates, significant damage in the male reproductive system is noticed [2,3]. What is more, chromates cause irritations and are cancerogenic [2,3]. Therefore, numerous regulations like REACH banning the application of chromates have been introduced. It made both academic and industrial researchers look for more environmentally friendly and user-safe, Cr(VI)-free alternatives [3].

In this review paper, the aim is to show and discuss the phenomenon of anion incorporation into anodic aluminum oxide and its impact on the corrosion performance of the formed coatings. Still, the problem of finding a green alternative for Cr(VI) anodizing has not been solved; thus, incorporation of corrosion inhibitors into the oxide might be one of the routes that could provide environmentally friendly coatings with corrosion performance competitive to the coatings formed using hazardous chromates.

## 2. Common Cr(VI)-Free Anodizing Alternatives

Legal regulations as well as awareness of the Cr(VI)-derived hazards made globally top-performing companies from developed countries look for appropriate type I anodizing alternatives and apply them in their facilities. One of the first commonly applied Cr(VI) alternatives was boric–sulfuric acid anodizing, applied in Boeing. Namely, Koop and Moji from Boeing Commercial Airplane Group, in 1992, reported anodizing of AA 2024 T3 and AA 7075 T6 in electrolytes containing 3–5 wt.% H_2_SO_4_ and 0.5–1.0 wt.% H_3_BO_3_ that resulted in a coating that withstood a 336 h (14 day) long salt spray test and fulfills Boeing’s standards for surface finishing and performance [4]. Unfortunately, this type of anodizing was re-evaluated by Thompson et al. in 1999: they anodized AA 7075 T6 at 15 V in electrolytes containing 45 g/L H_2_SO_4_ with H_3_BO_3_ concentrations ranging from 0 to 50 g/L and exposed the coated samples to neutral salt spray tests (NSST, 5% NaCl, ASTM B117 standard) for 7, 14, 60, and 90 days. They proved that chromic acid anodizing is still superior to BSAA—boric–sulfuric acid anodizing [5]. The same group studied the susceptibility of BSA-anodized 99.99% purity aluminum to pitting corrosion [6]. The electrolytes were composed of 45 g/L H_2_SO_4_ and 0 to 50 g/L H_3_BO_3_ (10 g/L was found to be optimum) and samples were anodized at 15 V. Pitting corrosion resistance was estimated as a time required to achieve current density oscillations, indicating pit formation and re-passivation under galvanic current density in 3.5 wt.% NaCl. Sulfuric acid-anodized samples and BSA-anodized samples were compared in terms of their pitting corrosion resistance, and the results obtained were found to be inconclusive: in some cases, sulfuric acid anodizing was found to provide better protection, while in other cases, BSAA was better. An important insight was delivered by Zhang et al.: they anodized AA 2024 at 18 V in an electrolyte containing not only boric (0.8 wt.%) and sulfuric acid (5 wt.%), but also phosphoric acid (50 g/L) and discovered that corrosion spots appeared after 216 h of exposure to the spray salt test, while for BSAA, the first corrosion traces appeared after 240 h [7]. Nevertheless, CAA was shown to provide the best corrosion performance. Domingues et al. reported anodizing of AA 2024 T3 in 15 wt.% H_2_SO_4_ with 0.5 M H_3_BO_3_ and 0.05 M Na_2_B_4_O_7_ and revealed that the fatigue resistance (based on an S-N plot) of the BSAA samples was comparable to that of CAA (difference of 2%) [8]. Additionally, the EIS (electrochemical impedance spectroscopy) data revealed that the temperature of the BSA bath has no impact on the protection of the coating.

Another quite common type I anodizing substituent is thin-film sulfuric acid anodizing (TFSAA). The main goal of this surface treatment is also to provide a corrosion-protective coating that is resistant to fatigue (i.e., the coating is not too thick and simultaneously provides satisfactory corrosion protection). Ding et al. reported the formation of TFSAA on AA 2024 T3 by anodizing in 10% wt. H_2_SO_4_ at 15 V (21 °C, 5–15 min). The formed coatings were compared to traditional type I, Cr(VI)-based anodizing (41.6–57.2 g/L chromic acid, 34 °C, 40 V, 35–45 min) [9]. In both cases, the samples were also sealed. The results showed that TFSAA results in a thin film (ca. 3 µm) and passed the 1350 h long pitting corrosion test. Nevertheless, it cannot be clearly stated that it could be an effective Cr(VI) anodizing substituent. In further experiments, Ding studied TFSAA (9.6% wt. H_2_SO_4_, 15 V, 21 °C, 5–20 min) on AA 2024 T3 and AA 6061 T6 [10]. Ding made an important contribution, especially from the industrial point of view. She reported that copper strengthening the alloy is also a great catalyst for the oxygen evolution reaction (OER); thus, much current is consumed in this reaction and, consequently, the current efficiency of alloy anodizing drops. AA 2024 has more Cu than AA 6061; thus, the oxide growth rate in the case of AA 2024 was much lower than in the case of AA 6061.

Another milestone approach was reported by del Olmo et al., where TFSAA was followed by the incorporation of corrosion inhibitor treatment, cerium salts, onto anodic alumina, to improve the corrosion performance of AA 2024 T3 [11]. Authors reported anodizing of AA2099-T83 aluminum extrusions and AA2060-T8E30 sheets in sulfuric acid (43 g/L, 15.0–15.5 V, 24–27 °C, 24–26 min) followed by immersion in the above-mentioned sealing bath. The anodizing was applied to the sealed elements and it was shown that the treatment was suitable for joining elements: no significant failures were found nor loss of adhesion reported.

One of the most commonly applied Cr(VI) anodizing alternatives is tartaric–sulfuric acid anodizing, also known as TSAA. Numerous researchers have reported outstanding properties of the coatings formed using mixtures of sulfuric and tartaric acid. The most exemplary and significant findings are gathered in Table 1. One of the first milestone papers about TSAA was published by Curioni et al., where they anodized AA 2024 T3 in 0.46 M sulfuric acid and 0.1 M ammonium pentaborate with 80 g/L (0.53 M) or 150 g/L (1 M) of tartaric acid [12]. The authors found that the presence of tartaric acid in the incorporated anodic alumina significantly decreased the anodic oxide dissolution rate; thus, the protected material was much less vulnerable to corrosion. Since then, much fundamental research on anodizing condition optimization has been delivered to the academic community by numerous researchers. For example, the impact of the chemical composition of the electrolyte in TSA anodizing on corrosion performance, assessed by neutral salt spray testing (NSST) and electrochemical impedance spectroscopy (EIS), was researched by Chen et al. [13]. They tested various concentrations of tartaric acid, ranging from 0 to 0.9 M in 0.4 M H_2_SO_4_, in fixed operating conditions, namely 14 V, 37 °C, and 1500 s. It was revealed that small additions of tartaric acid increased the susceptibility to corrosion of the AA 2099, while from the concentration of 0.53 M of tartaric acid in the electrolyte, a significant improvement in corrosion performance was noted. A similar study was delivered by Martinez-Viademonte et al. [14]. AA 2024 clad with AA 1050 was anodized using SA and TSA approaches and the obtained coatings were tested for adhesion. It was found that after TSA anodizing, the adhesion strength, measured via a peel test, improved ca. twofold when compared to SA anodizing. Already, at this point, it can be concluded that TSA provides better corrosion performance and adhesion than SA anodizing. Furthermore, Setianto and Korda studied the impact of the anodizing voltage on the corrosion performance of anodic coatings formed on AA 2024 T3 [15]. It was shown that anodizing in 44 g/L H_2_SO_4_ with 87 g/L C_4_H_6_O_6_ at 37 °C for 20 min can provide oxide coatings with varied corrosion performance: at 11 V, the corrosion potential, *E_corr_*, was as small as −0.721 V vs. Ag|AgCl, and the corrosion current density, *j_corr_*, equaled 3.32 × 10^−8^ A/cm^2^, while at 19 V, *E_corr_* was as high as −0.311 V vs. Ag|AgCl and *j_corr_* was 1.52 × 10^−8^. The results were more than promising when the TSA anodizing was compared to CA anodizing (46 g/L CrO_3_, 40 V, 40 °C, 40 min): *E_corr_* was reported to equal −0.555 V vs. Ag|AgCl and *j_corr_* was reported to equal 1.26 × 10^−7^ A/cm^2^. Usman et al. reported anodizing of AA 2024 T3 in 0.46 M H_2_SO_4_ + 0.533 M C_4_H_4_O_6_ at 7 and 14 V [16]. They anodized the alloy at two different voltages, 7 and 14 V, but for different times, 1500 and 3200–3300 s, to obtain an oxide layer with similar thickness, equal to ca. 33 µm. This approach allowed them to evaluate different pore morphologies with other unchanged features of the oxide. It is worth noting that pore diameter and interpore distance grow linearly with applied voltage [17]. The authors found their results inconclusive in terms of the impact of the pore size on the corrosion performance. On the one hand, the immersion tests indicated better corrosion performance of samples anodized at 7 V. On the other hand, neutral salt spray testing and electrochemical impedance spectroscopy indicated better corrosion performance of the oxide coatings grown at 14 V [16]. Interesting results were delivered by Raffin et al. [18]. They anodized AA 2024 T3 and AA 7075 T6/AA 7175 T6 in 40 g/L H_2_SO_4_ + 80 g/L C_4_H_4_O_6_ at 14 V for 25 or 45 min [18]. What is more, the samples subjected to anodizing were quite large, namely 100 mm per 120 mm. For shorter anodizing, the wetting contact angle (measured for high-purity water) was similar to bare samples, but when the anodizing time was extended, the wetting angle dropped. This phenomenon was caused by a commonly known effect: the longer the anodizing time, the greater the pore diameter, caused by longer exposure of the pores to the acidic environment and consequently, the longer the field-assisted etching [19]. What is more, the detailed EIS analyses showed that the longer the time, the greater the resistance caused by both the increased thickness of the porous layer and the greater thickness of the barrier layer, which shows the benefits of longer anodizing [18]. Additionally, the chemical composition of the oxide layer, provided by GD OES (Glow-Discharge Optical Emission Spectroscopy) showed the presence of the electrolyte anions as well as the alloying elements in the formed oxide layer [18]. Their findings in terms of the chemical composition of the anodic alumina formed on AA 2024T3 are similar to earlier reported findings by Iglesias-Rubianes et al. [20]. The authors of [20] showed the presence of Cu in anodic oxide formed on AA 2024 T3 anodized using the TSAA approach, using Rutherford backscattering spectroscopy (RBS).

Even more encouraging results are obtained when the anodized layer is sealed. In [21], AA 2024 T4 was reported to be anodized in 55 g/L H_2_SO_4_ with 88 g/L C_4_H_6_O_6_ at 14 V and 37 °C for 23 min, followed by various sealing procedures, including (i) HWS (hot water sealing: boiling in DI water for 30 min), (ii) nickel (II) fluoride sealing (4 g/L, followed by DI water treatment at 60 °C for 30 min), (iii) potassium dichromate (50 g/L, 90–95 °C, pH = 6.7, 30 min), or (iv) potassium permanganate with lithium nitrate and sodium molybdate (70 °C for 30 min). In all the cases, corrosion performance was reported to be improved. It is interesting that in all the cases, the corrosion current density dropped over 100 times, but only for the potassium permanganate with lithium nitrate and sodium molybdate sealing, the corrosion potential shifted significantly to a more noble value, by ca. 400 mV. EIS, NSST, and immersion tests confirmed that this sealing procedure provides coatings with the best corrosion performance. Already, at this point, it can be concluded that molybdenum salts are promising as a potential corrosion inhibitor of aluminum alloys, and when combined with anodizing, the corrosion performance of the coating is excellent. González-Rovira et al. reported anodizing of high-purity aluminum at various operating conditions: 0.41 M H_2_SO_4_ with 0.53 M C_4_H_6_O_6_ at 14 V and 37 °C for 20 min with a one-step approach and 25 V at 5–7 °C using a two-step approach (20 h first step and 1 h second step) [22]. As a reference, aluminum was anodized in 0.3 M H_2_SO_4_ at 25 V, 5–7 °C, also using a two-step approach (20 h first step, 1 h second step). The two-step approach employed chemical removal of the oxide grown in the first step and re-anodizing at the same voltage and electrolyte in the second step, according to the milestone paper by Masuda and Fukuda [23]. What González-Rovira et al. studied [22] is the impact of their approaches on the corrosion performance of anodic alumina without and with HWS (Table 1). The recorded corrosion current densities for the as-anodized samples were at the level of 10^−7^ A/cm^2^. After HWS, the lowest current density, below the detection limit, was recorded for samples anodized by the two-step approach using TSA. The results are complementary with the EIS data that show the highest charge transfer resistance for the same set of the samples.

The concept behind the application of tartaric acid as an additive to the electrolyte was the formation of water-insoluble aluminum tartrate. Researchers also apply other organic acids for the same reason. Aluminum citrate is also known as a compound with a very low solubility product; thus, citric–sulfuric acid anodizing also attracts the attention of anodizers (Table 2). 

Cabral-Miramontes et al. reported anodizing of AA 6061 in solutions containing 5 mL/L of H_2_SO_4_ + 210.12 g/L of citric acid or just sulfuric acid (199.04 g/L H_2_SO_4_) as a reference [24]. Anodizing was performed galvanostastically at 1.0 or 7.2 A/cm^2^, which is quite a large current density. The corrosion performance experiments indicated that the best corrosion performance was for the samples anodized using the CSA approach at the lower current density. The corrosion potential was as high as −0.343 V vs. Ag|AgCl.

Del Olmo et al. reported anodizing of AA 2024 T3 in a citric–sulfuric acid electrolyte [25]. When compared to other treatments, like TSA and TSAA, the |Z|_10mHz_ was much better for CSA. What is more, the application of Ce- and Li-salt-rich topping via a hybrid sol–gel approach increases the impedance of the coatings by an order of magnitude. Furthermore, the incorporation of the corrosion inhibitors, Ce and Li, was found to provide active corrosion protection. In [26], the authors anodized AA7150 in TSA with various amounts of titania sol, which allowed formation coatings with a thicker barrier layer and, consequently, better corrosion performance. The corrosion potential increased by over 200 mV, while the corrosion current density dropped over twofold.

A very meaningful contribution was delivered by Machado et al. [27], where AA 2024 T3 was anodized in 1.5 M H_2_SO_4_ with various organic acids, and a chronoamperometric study 30 mV above the alloy pitting potential was carried out as an indicator of the pitting corrosion susceptibility. To provide only one variable, all the anodizing was performed at 14 mA/cm^2^ and 37 °C for 25 min (according to the Airbus TSA standard) and the results were also referred to as standard type 1 anodizing, namely 40 g/L, 40 V, and 40 °C for 20 min. The chronoamperometry showed that the immunity to pitting corrosion grew within the following sequence: chromic acid > malic–sulfuric acid > tartaric–sulfuric acid > sulfuric acid without additives > citric–sulfuric acid > malonic–sulfuric acid > oxalic–sulfuric acid. A current density of 200 µA/cm^2^ was considered the pitting propagation current density.

Suzuki et al. reported the anodizing of high-purity Al in etidronic acid, C_2_H_8_O_7_P_2_ [28]. In their study, aluminum was anodized, using a two-step procedure in sulfuric acid (0.3 M, 298 K, 30 A/m^2^, 3 h), oxalic acid (0.3 M, 298 K, 30 A/m^2^, 3 h), citric acid (0.3 M, 298 K, 30 A/m^2^, 3 h), and etidronic acid (0.3 M, 298 K, 30 A/m^2^, 3 h), and the formed coatings were sealed using HWS (boiling water, 4 h). The corrosion performance was assessed using EIS and the authors noticed that due to the formation of a thicker barrier layer and anion incorporation, the corrosion performance was improved: alumina formed in etidronic acid withstood 2.5 M NaOH 10 times longer than alumina formed in sulfuric acid. After the sealing, the AAO formed in etidronic acid was also superior; thus, this previously unknown acid as an electrolyte might provide interesting insights into technical alloy anodizing in the future.

**Table 1:** Exemplary tartaric–sulfuric acid anodizing procedures.

Type of Material	Bath Composition	Anodizing Conditions	Corrosion Performance (Quantitative)			Remarks	Reference
			E_corr_/mV	J_corr_/A^.^cm^−2^	Other		
AA 2024 T3	0.46 M sulfuric acid and 0.1 M ammonium pentaborate with 80 g/L (0.53 M) or 150 g/L (1 M) of tartaric acid	14 V vs. SCE (3-electrode cell), RT ^1^	-	-	-	It was revealed that tartaric acid decreases the dissolution rate of the anodic oxide in the acidic environment.	[12]
AA 2099	0.4 M H_2_SO_4_	14 V, 37 °C, 1500 s	-	-	Since 0.53 M of tartaric acid NSST ^2^ shows significant improvement in corrosion performance.	Study of the impact of tartaric acid concentration in the anodizing bath on corrosion performance (EIS ^3^, NSST).	[13]
	0.4 M H_2_SO_4_ +0.1 M C_4_H_4_O_6_						
	0.4 M H_2_SO_4_ +0.3 M C_4_H_4_O_6_						
	0.4 M H_2_SO_4_ +0.53 M C_4_H_4_O_6_						
	0.4 M H_2_SO_4_ +0.7 M C_4_H_4_O_6_						
	0.4 M H_2_SO_4_ +0.9 M C_4_H_4_O_6_						
AA 2024 clad with AA 1050	40 g/L H_2_SO_4_	10 V, 37 °C, 20 min	-	-	-	The higher the electrolyte temperature, the better the adhesion of the film to a certain extent.	[14]
	40 g/L H_2_SO_4_ + 80 g/L C_4_H_4_O_6_	14 V, 45 °C, 20 min	-	-	-		
	40 g/L H_2_SO_4_ + 150 g/L C_4_H_4_O_6_	20 V, 55 °C, 20 min	-	-	-		
AA 2024 T3	44 g/L H_2_SO_4_ + 87 g/L C_4_H_6_O_6_	11 V, 37 °C, 20 min	−0.721	3.32 × 10^−8^	-	The impact of the anodizing voltage on corrosion performance was studied; corrosion potentials are given vs. Ag|AgCl.	[15]
		13 V, 37 °C, 20 min	−0.672	4.85 × 10^−8^	-		
		15 V, 37 °C, 20 min	−0.637	2.91 × 10^−9^	-		
		17 V, 37 °C, 20 min	−0.556	2.99 × 10^−9^	-		
		19 V, 37 °C, 20 min	−0.311	1.52 × 10^−8^	-		
		21 V, 37 °C, 20 min	−0.636	1.79 × 10^−6^	-		
	46 g/L CrO_3_	40 V, 40 °C, 40 min	−0.555	1.26 × 10^−7^	-		
AA 2024 T3	0.46 M H_2_SO_4_ + 0.533 M C_4_H_4_O_6_	14 V, 37 °C, 1500 s	-	-	-	Films with similar thickness (ca. 3 µm) but different pore morphologies were obtained; 7 V films have better performance in immersion tests, while 14 V has better performance in EIS and NSSTs.	[16]
		7 V, 37 °C, 3200–3300 s	-	-	-		
AA 2024 T3 AA 7075 T6 AA 7175 T6	40 g/L H_2_SO_4_ + 80 g/L C_4_H_4_O_6_	14 V, 37 °C, 25 or 45 min	-	-	-	Wetting angle and EIS study of samples anodized for different times; a semi-industrial 200 L bath with samples as big as 10 per 12 cm^2^ was used.	[18]
		7 V, 37 °C, 3200–3300 s	-	-	-		
AA 2024 T3	0.4 M H_2_SO_4_ + 0.53 M C_4_H_4_O_6_	5 mA/cm^2^ or 18 V, 308 K (35 °C), 1800 s	-	-	-	Rutherford backscattering spectroscopy (RBS) has proven that Cu from the alloy is incorporated into the anodic oxide.	[20]
AA 2024 T4	55 g/L H_2_SO_4_ + 88 g/L C_4_H_6_O_6_	14 V, 37 °C, 23 min	-	-	-	A comparative study of AA 2024 T4 TSA after various sealing is reported. Authors used sealing (1) in boiling DI water for 30 min (HWS); (2) in 4 g/L NiF_2_ at 25 °C for 30 min, followed by in DI water at 60 °C for 30 min; (3) in 50 g/L K_2_Cr_2_O_7_ (potassium dichromate) at 90–95 °C, pH = 6.7 (measured in 25 °C) for 30 min; and (4) in potassium permanganate, lithium nitrate and sodium molybdate at 70 °C for 30 min.	[21]
99.999% Al	0.41 M H_2_SO_4_ + 0.53 M C_4_H_6_O_6_	25 V, 5–7 °C, 20 h (1st step), 1 h (2nd step)	-	2.63 × 10^−7^; after sealing j_corr_ was below detection limit	1.20 × 10^5^	Two-step anodizing for corrosion protection was investigated; pores were sealed using boiling water (60 min).	[22]

^1^ RT—room temperature; ^2^ NSST—neutral salt spray test; ^3^ EIS—electrochemical impedance spectroscopy.

**Table 2:** Exemplary citric–sulfuric acid anodizing procedures.

Type of Material	Bath Composition	Anodizing Conditions	Corrosion Performance (Quantitative)			Remarks	Reference
			E_corr_/mV	J_corr_/A^.^cm^−2^	Other		
AA 6061	199.04 g/L H_2_SO_4_	1.0 A/cm^2^, 5 °C, 30 min	0.629	2.22 × 10^−8^	-	Anodizing was followed by HWS (95 °C, 30 min).	[24]
		7.2 A/cm^2^, 5 °C, 30 min	−0.566	5.93 × 10^−9^	E_pit_ = −0.297 mV		
	5 mL/L of H_2_SO_4_ + 210.12 g/L of citric acid	1.0 A/cm^2^, 5 °C, 30 min	−0.343	1.7 × 10^−8^	-		
		7.2 A/cm^2^, 5 °C, 30 min	−0.693	2.62 × 10^−6^	E_pit_ = −0.693 mV		
AA 2024 T3	1.5 M H_2_SO_4_	15 V, 25 °C,	-	-	-	EIS study of CSA, TSA and TSAA covered with Ce- and Li-rich hybrid sol–gel coatings was reported. Active corrosion protection was proven.	[25]
	1.5 M H_2_SO_4_ + 0.1 citric acid	15 V, 25 °C,	-	-	-		
	1.5 M H_2_SO_4_ + 0.25 M citric acid	15 V, 25 °C,	-	-	-		
	1.5 M H_2_SO_4_ + 0.50 M citric acid	15 V, 25 °C,	-	-	-		
AA 7150	45 g/L of H_2_SO_4_ + 8 g/L of citric acid	15 V, 25 °C, 20 min	−0.9827	8.86 × 10^−8^	-	Various amounts of Ti sol was added to the samples; the greater the amount of sol, the greater the corrosion potential and the lower the corrosion current density.	[26]
	45 g/L of H_2_SO_4_ + 8 g/L of citric acid + 25 mL/L Ti sol		−0.7652	3.36 × 10^−8^	-		

Another finding worth mentioning was reported by Yoganandan et al. [29]. They reported standard TSA anodizing (0.2 M H_2_SO_4_ + 0.5 M tartaric acid, 20 mA/cm^2^, 28 °C, 30 min) of AA 2024 T3 followed by sealing with 0.08 M KMnO_4_ and 0.04 M NH_4_VO_3_ (78 °C, 30 min). The corrosion current densities extracted from the polarization experiments revealed that the sealing is worth considering: the authors immersed TSA and TSA–sealed samples for 1 h and 2 weeks in 3.5% NaCl to study the degradation of the passive layer, and also in this case, high quality of the seal was confirmed. What is even more intriguing is that they also performed a 15-month-long outdoor test in Tamilnadu, India (tropical, hot, and moist climate), and no traces of corrosion were noticed on the sealed sample. These results suggest that d-electronic metals in high oxidation states are promising corrosion inhibitors for technical aluminum alloys.

For high-purity aluminum anodizing, already, rare electrolytes like acetylenedicarboxylic [30], arsenic [31] citric [32,33], croconic [34], glutaric [35], glycolic [36], malic [37,38], malonic [39,40,41], phosphonic [42], phosphonoacetic [43], pyrophosphoric [44,45,46], rhodizonic [34], selenic [47,48,49], squaric [50], or sulfamic acid [51] have been explored and proven to provide porous oxide. These can also be applied in aluminum technical alloy anodizing for corrosion protection; however, these have not been researched in this context yet, but some of them have been used in a mixed bath with sulfuric acid (i.e., [27]).

Therefore, there is still much potential in the research focused on Cr(VI)-free anodizing employing novel electrolytes accompanied by d-electronic metals in high oxidation states as the additives.

## 3. Incorporation of Anions into Anodic Alumina

Alumina formed by aluminum or technical aluminum alloy anodizing rarely includes pure Al_2_O_3_. Since the first broad, fundamental research was carried out in this field, traces of electrolyte species have been spotted. The development of analytical techniques allowed this phenomenon to be studied in detail. In 1966, Dorsey reported an IR spectroscopic study of anodic alumina formed in various electrolytes [52,53]. It was proven that the anodic alumina, depending on the applied electrolyte, had IR bands originating from the acidic groups belonging to the electrolyte, which confirms the incorporation of the electrolyte anions, such as sulfates or chromates, into the grown oxide layers. For example, anodizing in sulfuric or phosphoric acid allowed researchers to identify IR bands belonging to the sulfates and phosphates, respectively [52].

**Figure 3:**
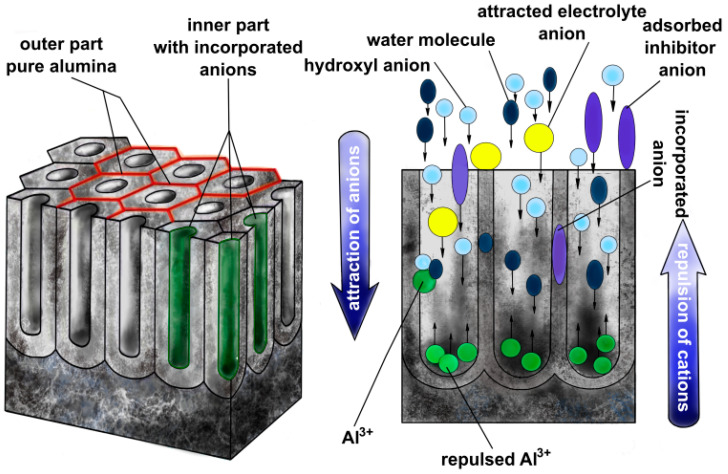
Scheme showing the inner and outer part of anodic alumina as well as the electrostatic forces interacting with ions in the electrolyte during anodization.

**Figure 4:**
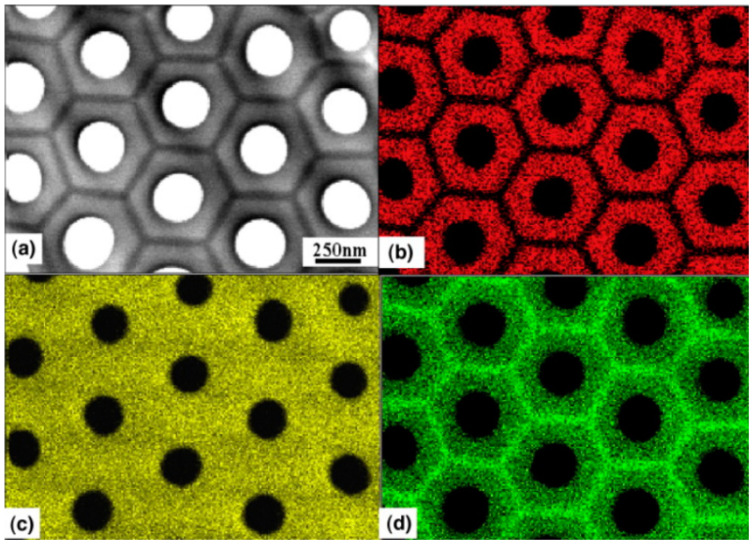
FEG-TEM top view (**a**) of anodic alumina formed in phosphoric acid and corresponding X-ray elemental maps of (**b**) phosphorus, (**c**) oxygen, and (**d**) aluminum, confirming incorporation of the phosphates into AAO. The duplex nature of the oxide is clearly seen, especially in the phosphorous map (**b**). Copied with permission from Elsevier from [56].

Davenport and Issacs studied the chemical composition of anodic alumina formed in 0.1 M K_2_CrO_4_ at 100 V via XANES (X-ray absorption near-edge spectroscopy) and revealed the presence of both chromates and Cr^3+^ species in the obtained anodic alumina [54]. This shows that anions not only incorporate in situ during anodizing by electrostatic attraction followed by adsorption (Figure 3), but may also undergo transformations, like reduction. Yamamoto and Baba reported important findings about the spatial distribution of the incorporated oxalates into anodic alumina, using Electron Spin Resonance (ESR) and infrared spectroscopy (IR) [55]. They revealed that the inner part of the oxide, closer to the pore center, is rich in the incorporated species, while the outer part is a rather pure alumina. This discovery from 1983 was confirmed experimentally much later, thanks to the development of elemental mapping with high-resolution transmission electron microscopy. For example, Le Coz et al. were some of the first to report elemental mapping of anodic alumina formed via anodizing in phosphoric acid, and earlier findings about the duplex structure were confirmed (Figure 4) [56]. Another classical and milestone paper was delivered by Parkhutik et al., where X-ray Photoelectron Spectroscopy (XPS) was employed to research the chemical composition of anodic alumina formed in sulfuric acid, chromic acid, and their mixtures [57]. It was proven that the acidic anions incorporate into the oxides via XPS, and also in their case, like in [54], both Cr(VI) and Cr(III) species were found in the anodic alumina. According to Vrublevsky et al. [39,40], aluminum anodizing in malonic and oxalic acid provides photoluminescence (PL) bands originating from those anions at 437 and 425 nm. Heat treatment of the anodic alumina with the incorporated anions induced changes in the PL bands; namely, when annealed at 600 °C, the PL intensity rises, while annealing at 700 °C decreases the intensity. Therefore, without any doubts, it was shown that the incorporation of the anions from the electrolyte may change the PL of the oxide, and heat treatment allows the properties to be controlled. Similar findings were revealed for the incorporation of tartrates [58]. Heat treatment of AAO with incorporated tartrates, from 500 to 700 °C, allowed an increase in the oxide band gap, which was tuned from 3.35 to 3.50 eV, while the as-anodized oxide had a band gap equal to 3.25 eV. It is also important to mention that the annealing of AAO with incorporated organic acid species transforms the incorporated anions into amorphous carbon. A milestone insight was provided by Thompson’s group and co-workers: they proved that all types of species can be incorporated into anodic alumina. A detailed overview paper by Thompson [59] shows that anions, like phosphates, neutral species like borates, and cationic species like tungsten ions can be successfully incorporated into anodic alumina, which provides numerous possibilities for tailoring the chemical composition of the oxides and, consequently, their properties. The simultaneous incorporation of a few types of anionic species was reported by Cantelli et al. [60,61]. The anodized aluminum in oxalic acid, phosphoric acid, and their mixtures found contributions in the PL spectra from both incorporated anions. What is more, the presence of the anionic species was confirmed by RBS (Rutherford backscattering spectrometry) [60,61].

**Figure 5:**
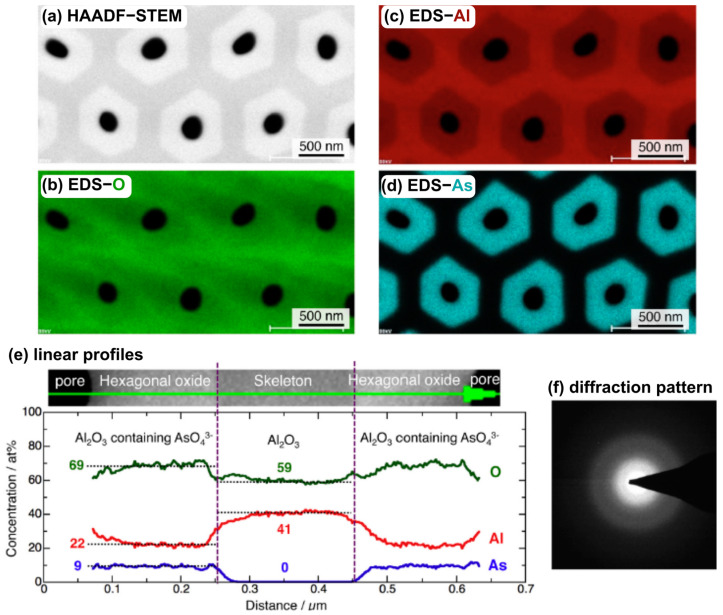
FEG-TEM top view (**a**) of anodic alumina formed in arsenic acid and corresponding X-ray elemental maps of (**b**) oxygen, (**c**) aluminum, and (**d**) arsenic, confirming incorporation of the arsenic anions into AAO, with EDS profile lines (**e**) and diffraction pattern (**f**). The duplex nature of the oxide is confirmed by the EDS profile (**e**). Copied with permission from Elsevier [31].

In recent years, due to the development of HR TEM and the accompanied increase in the resolution of elemental mapping, researchers started to directly show the duplex nature of the anodic alumina with incorporated anions. Kikuchi’s Group directly showed elemental maps, similar to those reported by Le Coz et al. (Figure Y [56]), of anodic alumina with incorporated anions like acetylenedicarboxylic [30], arsenic [31] (Figure 5) citric [32,33], croconic [34], glutaric [35], glycolic [36], malic [37,38], malonic [39,40,41], phosphonic [42], phosphonoacetic [43], pyrophosphoric [44,45,46], rhodizonic [34], selenic [47,48,49], squaric [50], or sulfamic acid [51]. In Figure YA, it is clearly seen that the incorporated arsenates are in the inner sphere of the oxide (close to the pore), while pure alumina builds the outer part, the skeleton of the oxide. The EDS maps are accompanied by the elemental analysis in the form of the profile lines, where it is even more apparent and supported with quantitative analysis (Figure 5e): in the outer part of the oxide, the concentration of As drops to zero, while in the inner part, it fluctuates around 9% at. Simultaneously, aluminum concentration in the inner part of the oxide is lower than in the outer part of the oxide and changes from ca. 59 to 69% at., respectively.

There is also a slight controversy linked to the incorporation of chromates into anodic alumina. As previously confirmed by Davenport and Issacs via XANES [54], Parkhutik et al. via XPS [56], and Stępniowski et al. [62] by EDS, no significant amount of chromates were found by HR TEM elemental mapping by Kikuchi et al. [63]. Kikuchi et al. anodized aluminum in 0.3 M chromic acid (40–160 V, 298–348 K), and others who detected chromates anodized aluminum in chromates at similar operating conditions, namely 20–50 V (0.3 M chromic acid, 20–50 °C) [62] or 100 V (0.1 M potassium chromate) [54]. Therefore, there are still unrevealed fundamental aspects of anion incorporation.

Glow-Discharge Optical Emission Spectroscopy (GDOES) is complementary to the above-mentioned HR TEM. This method’s greatest advantage is the ability to estimate the concentration of elements with low atomic numbers, like boron. Sato et al. have used GDOES to confirm the incorporation of borates and phosphate into AAO [64]. What is more, the authors [64] reported the incorporation of other anions into anodic alumina like citrates, succinates, adipates, tartrates, and salicylates. 

Apart from the impact of the incorporated anions on corrosion performance, it was found that incorporated anions change the properties of the oxides. Incorporated anions have a significant impact on properties of AAO like its capacitance [64], refractive index [65,66], or rate of chemical dissolution [67]. Currently, a mixture of a few acids is used to find optimum Cr(VI)-free corrosion performance, and simultaneously, few types of anions are incorporated into AAO. Bikulcius et al. reported anodization of AA 5052 in an electrolyte composed of sulfuric, oxalic, and citric acid that resulted in the formation of black coatings [68]. Furthermore, it was also shown that other additives to the electrolyte, like Cu(EDTA)_2_^4−^ coordination anions [69], or even organic molecules, like indigo carmine [70], can be successfully incorporated and change properties of the oxides, i.e., optical. Chernyakova et al. [71,72] have shown that anodizing high-purity Al in formic acid and formic acid with oxalic acid and molybdates facilitates the incorporation of carbon in the grown anodic alumina. The authors did not focus on the molybdate incorporation itself, but showed a strong impact of the molybdates on carbon incorporation.

## 4. Incorporation of Corrosion Inhibitors into Anodic Alumina

**Figure 6:**
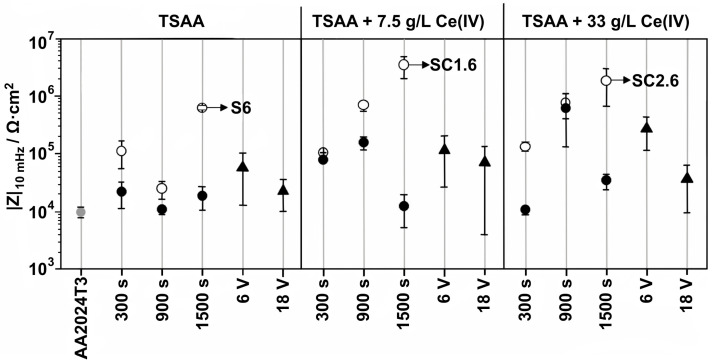
Scatter diagram of the impedance modulus, |Z| at 10 mHz for Ce(IV)-TSAA films. Filled and empty circles indicate oxide films developed at 15 mA/cm^2^ and 1.5 mA/cm^2^, respectively. Filled triangles indicate anodic films carried out under voltage-controlled mode (6 or 18 V). Adapted with permission from Elsevier [11].

As mentioned by Thompson [59], cations, anions, and ambient species can be incorporated into the growing anodic alumina. One of the most common corrosion inhibitors for aluminum alloys are cerium salts. Generally, cerium salts are usually used for sealing; e.g., after aluminum alloy anodizing, the nanoporous morphology is closed by immersion in an oxidative environment, and at this point, some corrosion inhibitors can be used to enhance the corrosion performance of the coating. For example, Selegård et al. reported anodizing of AA 2024 T3 in TSA at 14 V, after which the samples were immersed for 2 h in 5 g/L CeCl_3_ with 50 µL of 30% H_2_O_2_ followed by Hydrothermal Sealing (HTS) [73]. Obviously, the XPS analyses confirmed the presence of cerium, but the most remarkable result was the corrosion performance. The authors provided a facile immersion test in 5 wt.% NaCl that was up to 180 days long. After 180 days of immersion, no corrosion traces were found on samples sealed with cerium salts and HTS. However, samples that were subjected only to cerium (III) chloride immersion, without subsequent HTS, were corroded after 180 days. The poorest corrosion performance was noted for only anodized samples that were not post-treated. Similar research was reported by Ramirez et al. [74]. They anodized Al-clad AA 2024 T3 in TSA (40 g/L H_2_SO_4_ + 80 g/L C_4_H_6_O_6_, 14 V, 20 min, 37 °C) and subsequently immersed the samples in Ce(NO_3_)_3_ (50 mM, 2 min) with 10 vol.% H_2_O_2_ at different temperatures (25, 50, 75 °C). EDX, RBS, XPS, and GDOES experiments confirmed the presence of cerium in the oxide after pore sealing. Corrosion performance was assessed using EIS (0.1 and 0.5 M NaCl, up to 1680 h/70 days) and it was found that Ce^3+^ sealing at 25 and 50 °C provides better corrosion performance than sealing at higher temperatures. Thus, the advantage of the presence of cerium is obvious. Del Olmo et al. [11] anodized AA 2024 T3 using 150 g/L of H_2_SO_4_ enriched in cerium salts, namely 7.5 or 33.0 g/L Ce(SO_4_)_2_ at either constant current density, 1.5 or 15.0 mA/cm^2^, or constant voltage, 6 or 18 V. For comparison, CAA samples were provided by a commercial partner (MTU Aeroengines). Figure 6 shows a scatter diagram where corrosion performance screening of various combinations of treatments is compared using |Z| at 10 mHz. Already, at this point, it is apparent that the best treatment is galvanostatic anodizing at 15 mA/cm^2^ for 1500 s, and the addition of Ce^4+^ salt improves the corrosion performance significantly. 

Thus, the effect of the incorporated corrosion inhibitor into the anodic oxide is noticeable, and the presence of cerium was also confirmed by RBS. The EIS study (1 h and 28 days in 3.5 wt.% NaCl) revealed partial loss of the protection effect after 28 days (|Z|_10mHz_ dropped); however, cerium cation incorporation still provided better corrosion protection than standard TSA samples. Furthermore, the NSST (5 wt.% NaCl at 6.5–7.2 pH range, 35 °C, for 1000 h; painted and scratched samples) was performed for samples anodized with 7.5 g/L cerium (IV) sulfate and compared with the CAA sample, and unfortunately, CAA still seems to be superior in this type of testing. The corrosion study was also accompanied by adhesion and fatigue testing, which is crucial. There is always a risk that a coating with satisfying corrosion performance might have poor mechanical performance, which disqualifies it from further development in real-life applications. The paint adhesion test revealed satisfactory performance, while the fatigue testing showed (S-N curves) that bare AA 2024 T3 withstands more cycles (300 MPa after 10^7^ cycles) under greater load than when anodized (265 and 256 MPa after 10^7^ cycles for samples anodized in TSA with Ce^4+^ and CAA reference, respectively).

Of course, natural substituents of Cr(IV)-based anodizing techniques are the ones employing other, d-electronic metals on high oxidation states, in the forms of anions. Permanganates are a great example of such an approach. Mohammadi et al. reported anodization of AA 2024 T3 in 17 wt.% H_2_SO_4_ at 20 V for 30 min with NH_4_H_2_PO_4_ (50 or 100 mM) or KMnO_4_ (50 or 100 mM) as additives [75]. Extended corrosion study allowed an understanding of the impact of the incorporated corrosion inhibitors on the anodic coatings’ performance (Table 3). EDS confirmed the incorporation of the anions, and the corrosion study left no doubt about the improvement of the performance of the anodic coating: in the case of NH_4_H_2_PO_4_, E_corr_ reached up to −550 mV vs. Ag|AgCl (50 mM), and the corrosion current density was as low as 2.5 × 10^−8^ A/cm^2^ (100 mM), while for anodizing without the additive, the *E_corr_* equaled −633 mV vs. Ag|AgCl and the current density equaled 2.23 × 10^−6^ A/cm^2^. A similar but slightly worse effect was achieved for permanganates: *E_corr_* raised to −543 mV vs. Ag|AgCl, while the corrosion current density dropped to 1.34 × 10^−7^ A/cm^2^. Valuable information was also provided by the EIS study. When the resistance of the barrier layer (*R_b_*) and capacitance of the CPE (imperfect capacitor) (*c_b_*) are taken under consideration, a significant increase in the resistance, accompanied by a significant drop in capacitance, will be noticed (Table 3), which indicates the formation of the thicker barrier layer for samples anodized in baths with corrosion inhibitors [75]. 

Also, Mohammadi et al. reported anodizing of AA 2024 T3 in 17 wt.% sulfuric acid with various amounts of KMnO_4_, ranging from 10 mM to 250 mM, and found a strong inhibition effect, acknowledged to be due to the permanganates’ incorporation into the formed anodic alumina [76]. It was revealed that the greater the amount of the inhibitor, the higher the corrosion potential (increasing from −0.633 for anodizing without inhibitor to −0.531 V vs. Ag|AgCl for sample anodized with 250 mM of KMnO_4_) and the lower the corrosion current density (dropped from 2.23 × 10^−6^ A/cm^2^ for sample anodized in bath without inhibitor to 2.6 × 10^−8^ for samples anodized in electrolyte containing 250 mM). The results were supplemented by an EIS study, where a barrier layer resistance increase was accompanied by a barrier layer capacitance drop (Table 3). Moutarlier et al. reported an extended EIS study of AA 2024 T3 anodizing in sulfuric acid with potassium permanganate (Table 3) and sodium molybdate (Table 4) [77]. The barrier layer resistance (*R_b_*) and capacitance (*c_b_*), as well as the exponent of the constant phase element, CPE (*α*), were considered as a measure of corrosion performance. The extended study, employing immersion of the anodized alloy in 5 wt.% NaCl for 1, 2, 10, 24, and 48 h, showed the rate of coating degradation and a simultaneous strong inhibition effect (Table 3 and Table 4). The as-anodized samples showed quite rapid degradation, i.e., the capacitance of the barrier layer grew, which signifies its thinning (the thinner the dielectric layer in the capacitor, the greater the capacitance of the capacitor). For samples anodized in pure sulfuric acid, the capacitance of the barrier layer (*c_b_*) increased from 0.6 (1 h) to 10.2 µF/cm^2^ (48 h), while for samples anodized with potassium permanganate, the capacitance of the barrier layer grew from 0.5 (1 h) to 7.8 µF/cm^2^ (48 h), which translates into hindered degradation of the barrier layer (Table 3). These results are supplemented by a simultaneous drop in the CPE exponent, α, from 0.94 to 0.82 and from 0.98 to 0.87 for coatings formed in sulfuric acid and sulfuric acid with potassium permanganate, respectively. Additionally, the resistance of the barrier layer, *R_b_*, dropped from ca. 5 × 10^7^ Ω·cm^2^ for both to ca. 10^3^ Ω·cm^2^ and 10^4^ Ω·cm^2^ for coatings formed in sulfuric acid and sulfuric acid with potassium permanganate, respectively. Thus, a strong inhibition effect due to the presence of the incorporated permanganates is noticed. The inhibition effect is even stronger when the anodized samples are subjected to hot water sealing (HWS; boiling DI water for 30 min): the capacitance of the barrier layer drops from 0.5 (1 h) to 3.9 µF/cm^2^ (48 h) and from 0.2 (1 h) to 2.4 µF/cm^2^ (48 h) for samples anodized without and with KMnO_4_. Similarly, there is a strong impact of the anodizing bath on α, which drops for sealed samples from 0.94 to 0.82 (no inhibitor) and from 0.98 to 0.87 (bath with KMnO_4_). Additionally, *R_b_* dropped from ca. 5 × 10^7^ Ω·cm^2^ for both to ca. 10^3^ Ω·cm^2^ and 10^7^ Ω·cm^2^ for coatings, immersed for 48 h in 5% wt. NaCl, formed in sulfuric acid and sulfuric acid with potassium permanganate, respectively. This shows a strong enhancement of the inhibition effect provided by the incorporated permanganates.

Moutarlier et al.’s research also provided significant insights into the application of molybdates as corrosion inhibitors [77]. The inhibition effect of molybdates introduced into the anodic alumina was also significant; however, it was weaker than in the case of potassium permanganate. AA 2024 T3 anodizing in 17 wt.% H_2_SO_4_ with 0.1 M Na_2_MoO_4_ allowed anodic coatings to be obtained, with the capacitance of the barrier layer increasing from 0.6 to 9.8 µF/cm^2^ (48 h), and after HWS, it increased from 0.5 (1 h) to 3.4 µF/cm^2^ (48 h in 5 wt.% NaCl) (Table 4). Simultaneously, *R_b_* dropped from ca. 5 × 10^7^ Ω·cm^2^ to ca. 10^3^ Ω·cm^2^ and to ca. 5 × 10^5^ Ω·cm^2^ for the as-anodized and HWS samples, respectively. In general, it is important to note that molybdates, due to their unusual chemistry, have great potential as corrosion inhibitors for aluminum alloys. Kwolek reported a very detailed study on the chemistry of molybdates as AA 7075 T6 corrosion inhibitors [78]. In solutions with low pH, like electrolytes applied for aluminum anodizing, the simple molybdate anions tend to condensate and form complex oligomolybdates, i.e., [Mo_7_O_24_]^6−^, [Mo_36_O_112_(H_2_O)_16_]^8−^, or even [Mo_154_(NO)_14_O_420_(OH)_28_(H_2_O)_70_]^25−^ [78]. Heteropolyoxomolybdates tend to form when other elements, e.g., acidic anions in the anodizing electrolytes, are present. Kwolek has proven that the presence of molybdates in the electrolyte improves the corrosion performance of aluminum alloys, which has been confirmed by independent methods, including gravimetry, microscopy, polarization, and EIS. For the optimized concentration of molybdates in phosphoric acid, it was proven that the corrosion rate drops 23 times when compared to electrolytes without molybdates. It was shown that heteropolyoxomolybdates are formed, adsorbed on the alloy surface, and hamper aluminum oxidation. Moreover, it was also found that cathodic processes, on the more noble phases of the alloy, are also hindered to a certain extent. Lopez-Garrity and Frankel considered molybdates as the corrosion inhibitors of AA 2024 T3 [79]. The noticed a significant improvement in the corrosion performance when 0.1 M of Na_2_MoO_4_ was added to the electrolyte, 0.1 M NaCl. Pitting corrosion was shifted by 250 mV to more noble values. The corrosion inhibition mechanism probably involves a reduction of MoO_4_^2−^ species on the surface to MoO(OH)_2_ over the alloying element-rich intermetallic phases, hampering the cathodic reaction of the corrosive cell and lowering the corrosion rate. Additionally, the total impedance, in the presence of the molybdates, was 40 times greater than in the reference, Mo-free samples. It is important to mention, that improvements were not noted for the deaerated electrolytes, which means that molybdates are weak oxidants when compared to chromates or permanganates. In [80], Moutarlier et al. reported AA 2024 T3 anodizing using the SAA approach with 0.1 M Ce(SO_4_)_2_ or 0.1 M Na_2_MoO_4_. Despite both compounds being known as good corrosion inhibitors, some opposite effects were noticed. Anodizing in Ce-rich electrolytes allowed the recorded voltage to decrease and a thicker oxide to form, while anodizing in a Mo-enriched electrolyte resulted in a greater recorded voltage, and a thinner oxide formed (Table 4). The Mo effects are probably linked to the fact that in an acidic electrolyte, condensation of molybdates occurs, which translates into bigger and less mobile charge carriers. Probably, a thicker barrier layer at the bottom of the anodic alumina pores might also form. Also, Moutalier et al. reported a comparative study of AA 2024 T3 anodizing in 150 g/L H_2_SO_4_ without and with various additives, including 0.01 M KMnO_4_ (Table 3), 0.01 M Na_2_MoO_4_ (Table 4), and 0.01 M Na_2_Cr_2_O_7_ [81]. The GDOES study confirmed the successful incorporation of Mo and Mn species into the anodic alumina. The inhibitor effect was also significant: for example, the pitting corrosion potential shifted from −400 mV (SAA anodizing) to −250 mV and −90 mV vs. Ag|AgCl for anodizing with 0.01 Na_2_MoO_4_ and 0.01 M KMnO_4_, respectively, and in the case of permanganates, it exceeds results for CAA, where *E_pit_* = −120 mV vs. Ag|AgCl. The EIS study allowed the authors to estimate the charge transfer resistance and capacitance of the barrier layer and also showed impactful results for the applied corrosion inhibitors. Namely, the charge transfer resistance increased from 4100 Ω·cm^2^ for SAA to 8500 and 16,000 Ω·cm^2^ for anodizing with 0.01 Na_2_MoO_4_ and 0.01 M KMnO_4_, respectively, and again in the case of permanganates, it exceeds results for CAA, where *R_p_* = 15,300 Ω·cm^2^. Surprisingly, the lowest value of the barrier layer capacitance, translating into the thickest barrier layer, was for CAA (0.10 µF/cm^2^), while for anodic alumina formed in 0.01 Na_2_MoO_4_ and 0.01 M KMnO_4_, it equaled 0.28 and 0.19 µF/cm^2^, respectively. This research shows that permanganates have great potential as corrosion inhibitors, providing coatings with corrosion performance comparable to the CAA coatings. Nevertheless, significant inhibition was also provided by molybdates. In further research, Moutalier et al. investigated the impact of Na_2_MoO_4_ concentration in the anodizing bath on the corrosion performance of anodic alumina [82]. For the highest concentration of Na_2_MoO_4_, 0.5 M, the pitting potential shifted from −400 to 0 mV vs. Ag|AgCl, when compared to SAA. Significant improvements were also noticed when EIS was applied to characterize the coatings’ corrosion performance: barrier layer resistance and capacitance have proven corrosion inhibition. The resistance grew from 3.5 × 10^7^ Ω·cm^2^ (no inhibitor added) to 8 × 10^7^ Ω·cm^2^ (0.5 M Na_2_MoO_4_), while the capacitance dropped from 1.01 µF/cm^2^ (no inhibitor added) to 0.54 µF/cm^2^ (0.5 M Na_2_MoO_4_). Simultaneously, for reference, the CAA sample’s barrier layer resistance and capacitance equaled 3 × 10^8^ Ω·cm^2^ and 0.11 µF/cm^2^, respectively; nevertheless, the pitting potential was lower (−50 mV vs. Ag|AgCl) than for samples anodized in sulfuric acid with 0.5 M Na_2_MoO_4_. Thus, it shows that for corrosion performance comparable to CAA, a concentration of molybdates, as high as 0.5 M, has to be applied in the sulfuric acid anodizing bath. Very insightful chemical composition analysis of the oxide was delivered using AAS (Atomic Absorption Spectroscopy) of anodic alumina formed on AA 2024 T3 without and with the addition of 0.1 and 0.5 M Na_2_MoO_4_ in sulfuric acid [83]. It was shown quantitatively that for 0.1 and 0.5 M Na_2_MoO_4_, 0.16 µg/cm^2^ and 0.96 µg/cm^2^ of Mo were detected, respectively. Thus, the incorporation of an anionic corrosion inhibitor was quantified for the first time. The data were supplemented by GDOES and XPS studies. Interesting results were reported in [84]. Usually, sulfuric acid is used as the base electrolyte for further modifications with the additives, while in this case, sodium molybdates, in various amounts, were added to 50 g/L Na_2_B_4_O_7_·10H_2_O. It was found that there is a certain, optimum sodium molybdate concentration, 0.3–0.4 M, where the greatest amount of the molybdates is incorporated, and consequently, the best corrosion performance, e.g., the highest pitting potentials, is achieved.

**Table 3:** Gathered corrosion performance of aluminum alloys subjected to anodizing with permanganates as inhibitors.

Type of Material	Bath Composition	Anodizing Conditions	Corrosion Performance (Quantitative)			Remarks	Reference
			E_corr_/mV	J_corr_/A^.^cm^−2^	Other		
AA 2024 T3	17 wt.% H_2_SO_4_	20 V, 30 min, RT	−0.633	2.23 × 10^−6^	R_b_ = 4295 Ω *C_b_* = 37.9 µF	-	[75]
	17 wt.% H_2_SO_4_ + 50 mM KMnO_4_		−0.555	4.11 × 10^−7^	R_b_ = 9027 Ω *C_b_* = 20 µF	0.46 at. % (EDS) of Mn	
	17 wt.% H_2_SO_4_ + 100 mM KMnO_4_		−0.543	1.34 × 10^−7^	R_b_ = 27,200 Ω *C_b_* = 8.88 µF	0.82 at. % (EDS) of Mn	
	17 wt.% H_2_SO_4_ + 50 mM NH_4_H_2_PO_4_		−0.550	5.8 × 10^−8^	R_b_ = 27,900 Ω *C_b_* = 4.71 µF	2.61 at. % (EDS) of P	
	17 wt.% H_2_SO_4_ + 100 mM NH_4_H_2_PO_4_		−0.587	2.5 × 10^−8^	R_b_ = 116,000 Ω *C_b_* = 2.28 µF	4.25 at. % (EDS) of P	
AA 2024 T3	17 wt.% H_2_SO_4_	20 V, 30 min, RT	−0.633	2.23 × 10^−6^	R_b_ = 4295 Ω *C_b_* = 37.9 µF	The impact of potassium permanganate additive to the sulfuric acid was investigated; however, no chemical composition examinations of the anodic film were shown. Nevertheless, a strong inhibition effect was noticed.	[76]
	17 wt.% H_2_SO_4_ + 0.01 M KMnO_4_		−0.578	1.23 × 10^−6^	R_b_ = 5526 Ω *C_b_* = 34 µF		
	17 wt.% H_2_SO_4_ + 0.05 M KMnO_4_		−0.555	4.11 × 10^−7^	R_b_ = 9027 Ω *C_b_* = 20 µF		
	17 wt.% H_2_SO_4_ + 0.10 M KMnO_4_		−0.543	1.34 × 10^−7^	R_b_ = 27,200 Ω *C_b_* = 8.88 µF		
	17 wt.% H_2_SO_4_ + 0.25 M KMnO_4_		−0.531	2.6 × 10^−8^	R_b_ = 148,000 Ω *C_b_* = 2.94 µF		
AA 2024 T3	17 wt.% H_2_SO_4_	1 mA/cm^2^, 20 min, 20 °C	After anodizing, samples were immersed in 5 wt.% NaCl (35 °C) for 1, 2, 10, 24, and 48 h and EIS spectra were recorded. The capacitance of the barrier layer (*c_b_*) increased from 0.6 (1 h) to 10.2 µF/cm^2^ (48 h). After hot water sealing, *c_b_* ranged from 0.5 (1 h) to 3.9 µF/cm^2^ (48 h).				[77]
	17 wt.% H_2_SO_4_ + 0.1 M KMnO_4_		The capacitance of the barrier layer grew from 0.5 (1 h) to 7.8 µF/cm^2^ (48 h). After hot water sealing, *c_b_* ranged from 0.2 (1 h) to 2.4 µF/cm^2^ (48 h).				
AA 2024	150 g/L H_2_SO_4_	15–20 V, 20 °C,	E_pit_ =−400 mV vs. Ag|AgCl, R_po_ = 4100 Ω·cm^2^, *c_b_* = 0.46 µF/cm^2^.			HWS for 30 min; GD OES confirmed the presence of Mn in anodic alumina.	[81]
	150 g/L H_2_SO_4_ + 0.01 M KMnO_4_		E_pit_ = −90 mV vs. Ag|AgCl, R_po_ = 16,000 Ω·cm^2^, c_b_ = 0.19 µF/cm^2^.				

**Figure 7:**
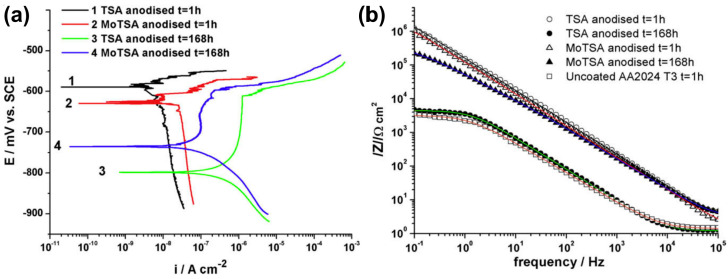
Polarization curves (**a**) and impedance modulus (**b**) of AA 2024 T3 subjected to anodizing with TSA and MoTSA approach. Samples were immersed in 3 wt.% NaCl for 1 or 168 h. Copied with permission from Elsevier [85].

Molybdates have one great advantage over permanganates: these compounds, due to being slighter oxidants, can be applied along with organic acids, which opens the door to a new class of anodizing baths. Organic compounds, like citric or tartaric acids, are easily oxidized by permanganates, which can be easily observed by decolorization of the mixture of KMnO_4_ with the mentioned acids, especially at low pH (acidic pH < 7). One of the first milestone papers, where the TSA approach was enriched with molybdates, was reported by García-Rubio et al. [85]. At first glance, it seemed that TSA provides slightly better corrosion performance than MoTSA, molybdate-enriched TSA, when samples after 1 h of immersion in 3 wt.% NaCl were investigated (Table 4). The corrosion and pitting potentials were greater for the TSA samples than for the MoTSA samples, and the corrosion current density of the TSA samples was smaller than for the MoTSA sample. EIS-derived capacitances of the barrier layer were comparable. The situation looks different when samples are immersed for 168 h in 3 wt.% NaCl: the corrosion potential for samples anodized with MoTSA is greater than for the TSA samples (Figure 7a). Already, the polarization curves provide information on how incorporated molybdate improved corrosion performance after 168 h of immersion: *E_corr_* was −735 mV vs. SCE (vs. −798 mV vs. SCE for TSA sample), *j_corr_* = 132 nA/cm^2^ (vs. 1590 nA/cm^2^ for TSA samples), and *E_pitt_* was −598 mV vs. SCE (vs. −611 mV vs. SCE for TSA sample). Moreover, EIS analysis also confirms much better corrosion performance of the MoTSA samples (Figure 7b). It can be seen on the Bode plot where the impedance modulus, |Z|, at low frequencies, is ca. 50 times greater for MoTSA after 168 h of immersion than for TSA after 168 h of immersion. Moreover, detailed analysis based on the equivalent circuits shows ca. eight-times smaller barrier layer capacitance of the MoTSA sample than the TSA sample (3.87 µF/cm^2^ vs. 30.3 cm^2^). It means that samples with incorporated molybdates degrade much slower than the TSA samples. Therefore, the inhibition effect provided by the incorporated molybdates is significant and sodium molybdate is a valuable modifier of the TSA approach. The authors [85] also performed NSST tests in which the MoTSA samples were also found to be much more corrosion-resistant than the TSA samples. 

**Figure 8:**
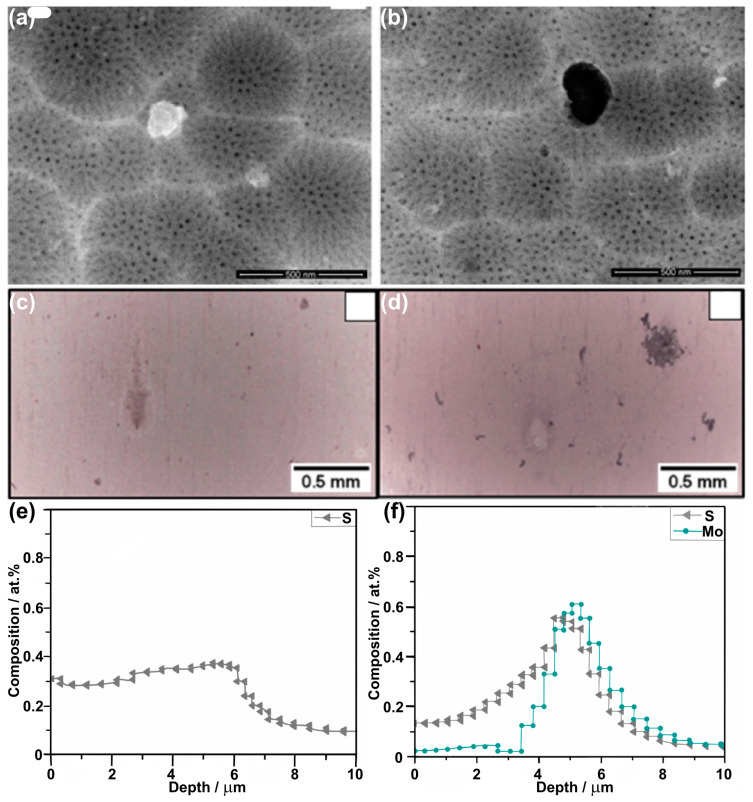
FE-SEM top-view images (**a**,**b**), optical top-view images of samples immersed for 77 weeks (**c**) and 86 weeks in 0.5 M NaCl (**d**), and RBS profiles (**e**,**f**) of AA 2024 T3 after TSA (**a**,**c**,**e**) and MoTSA (**b**,**d**,**f**) anodizing. Copied and adapted with permission from Elsevier [86].

**Figure 9:**
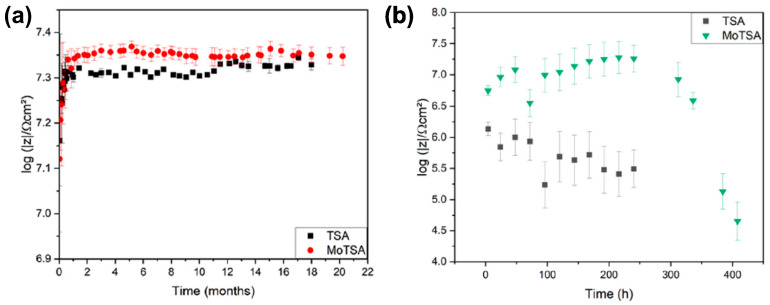
Evolution of low-frequency impedance modulus, |Z|, over time for TSA and MoTSA samples in 0.5 M NaCl (**a**) and acidified 0.1 M NaCl (pH = 4) (**b**). Copied with permission from Elsevier [86].

De Almeida et al. [86] also reported TSA and MoTSA on AA 2024 T3 (Table 4), and the incorporation of molybdates was confirmed by numerous methods, including XPS, RBS, and STEM-HAADF. It was also shown that an introduction of molybdates into the electrolyte does not affect the nanoporous morphology of the oxide (Figure 8a,b). Already, macroscopically, it could be noticed that the corrosion performance of MoTSA is better than TSA; similar corrosion damages were observed much later for MoTSA samples (86 vs. 77 weeks in 0.5 M NaCl; Figure 8c,d). The authors also quantitatively proved by RBS that up to 0.6 at% of Mo was incorporated into the anodic oxide (Figure 8f) and it was always accompanied by sulfur, co-incorporated during the anodizing (Figure 8e,f). Despite the authors having shown the incorporation of molybdates (Mo^VI+^) via XPS, it was shown that it tends to slightly overlap with sulfur species due to a similar binding energy range. Very informative results were reported by long-lasting EIS experiments, where |Z| at low frequencies was considered as a quantitative measure of the corrosion performance (Figure 9b). In 0.5 M NaCl, no significant change in |Z| was noticed even after 22 months of immersion; however, greater values were measured for MoTSA samples (Figure 9a). When the immersion was performed in 0.1 M NaCl but in a solution with pH as low as 4, a strong degradation of anodic films was noticed. The TSA was much less resistant to corrosion than MoTSA; namely, for the TSA samples, |Z| gradually decreased, while the |Z| of the MoTSA samples was firstly slightly raised and dropped to the TSA level after 384 h of immersion (TSA immersion was terminated after 240 h). Thus, without any doubt, a strong inhibition effect of molybdates was proven.

**Table 4:** Gathered corrosion performance of aluminum alloys subjected to anodizing with molybdates as inhibitors.

Type of Material	Bath Composition	Anodizing Conditions	Corrosion Performance (Quantitative)		Remarks	Reference
AA 2024 T3	17 wt.% H_2_SO_4_	1 mA/cm^2^, 20 min, 20 °C	After anodizing, samples were immersed in 5 wt.% NaCl (35 °C) for 1, 2, 10, 24, and 48 h and EIS spectra were recorded. The capacitance of the barrier layer (*c_b_*) increased from 0.6 (1 h) to 10.2 µF/cm^2^ (48 h). After hot water sealing, *c_b_* ranged from 0.5 (1 h) to 3.9 µF/cm^2^ (48 h).			[77]
	17 wt.% H_2_SO_4_ + 0.1 M Na_2_MoO_4_		The capacitance of the barrier layer grew from 0.6 (1 h) to 9.8 µF/cm^2^ (48 h). After hot water sealing, *c_b_* ranged from 0.5 (1 h) to 3.4 µF/cm^2^ (48 h).			
AA 2024 T3	150 g/L H_2_SO_4_ + 0.1 M Na_2_MoO_4_	1.0 or 1.5 mA/cm^2^, 20 min, 20 °C	When Mo-containing electrolyte was used, greater voltage was recorded and thinner oxide was formed.			[80]
AA 2024	150 g/L H_2_SO_4_	15–20 V, 20 °C,	E_pit_ = −400 mV vs. Ag|AgCl, R_po_ = 4100 Ω·cm^2^, c_b_ = 0.46 µF/cm^2^.		HWS for 30 min; GD OES confirmed the presence of Mo in anodic alumina.	[81]
	150 g/L H_2_SO_4_ + 0.01 M Na_2_MoO_4_		E_pit_ = −250 mV vs. Ag|AgCl, R_po_ = 8500 Ω·cm^2^, c_b_ = 0.28 µF/cm^2^.			
AA 2024 T3	150 g/L H_2_SO_4_	1.0 mA/cm^2^, 20 min, 20 °C	E_pit_ = −400 mV vs. Ag|AgCl, R_b_ = 3.5 × 10^7^ Ω·cm^2^, c_b_ = 1.01 µF/cm^2^.		HWS for 30 min.	[82]
	150 g/L H_2_SO_4_ + 0.01 M Na_2_MoO_4_		-			
	150 g/L H_2_SO_4_ + 0.1 M Na_2_MoO_4_		E_pit_ = −300 mV vs. Ag|AgCl, R_b_ = 4 × 10^7^ Ω·cm^2^, c_b_ = 0.86 µF/cm^2^.			
	150 g/L H_2_SO_4_ + 0.5 M Na_2_MoO_4_		E_pit_ = 0 mV vs. Ag|AgCl, R_b_ = 8 × 10^7^ Ω·cm^2^, c_b_ = 0.54 µF/cm^2^.			
AA 2024 T3	1.5 M H_2_SO_4_	1.0 mA/cm^2^, 20 min, 20 °C	C_b_ = 0.62 µF/cm^2^	Atomic Absorption Spectroscopy (AAS) results have shown that anodizing with 0.1 M and 0.5 M Na_2_MoO_4_ provides 0.16 µg/cm^2^ and 0.96 µg/cm^2^ of Mo into anodic alumina. XPS and GDOES also confirmed the presence of Mo.		[83]
	1.5 M H_2_SO_4_ + 0.1 M Na_2_MoO_4_		C_b_ = 0.60 µF/cm^2^			
	1.5 M H_2_SO_4_ + 0.5 M Na_2_MoO_4_		C_b_ = 0.58 µF/cm^2^			
AA 2024 T3	50 g/L Na_2_B_4_O_7_·10H_2_O	40 V, 70 °C, 60 min, pH = 10 (NaOH)	The maximum amount of Mo was incorporated when the alloy was anodized in an electrolyte containing 0.3 M Na_2_MoO_4_ (EDS). The highest values of pitting potentials were for samples anodized in electrolytes containing 0.3 and 0.4 M Na_2_MoO_4_			[84]
	50 g/L Na_2_B_4_O_7_·10H_2_O + 0.1 M Na_2_MoO_4_					
	50 g/L Na_2_B_4_O_7_·10H_2_O + 0.2 M Na_2_MoO_4_					
	50 g/L Na_2_B_4_O_7_·10H_2_O + 0.3 M Na_2_MoO_4_					
	50 g/L Na_2_B_4_O_7_·10H_2_O + 0.4 M Na_2_MoO_4_					
	50 g/L Na_2_B_4_O_7_·10H_2_O + 0.5 M Na_2_MoO_4_					
AA 2024 T3	0.53 M C_4_H_6_O_6_ + 0.46 M H_2_SO_4_	14 V, 37 °C, 20 min	1 h immersion in 3 wt.% NaCl: E_corr_ = −594 mV vs. SCE, j_corr_ = 8 nA/cm^2^, E_pitt_ = −569 mV vs. SCE, c_b_ = 0.66 µF/cm^2^ 168 h immersion in 3 wt.% NaCl: E_corr_ = −798 mV vs. SCE j_corr_ = 1590 nA/cm^2^, E_pitt_ = −611 mV vs. SCE, c_b_ = 30.3 µF/cm^2^		RBS and GDOES confirmed the presence of incorporated sulfates.	[85]
	0.53 M C_4_H_6_O_6_ + 0.46 M H_2_SO_4_ + 0.25 M Na_2_MoO_4_		1 h immersion in 3 wt.% NaCl: E_corr_ = −627 mV vs. SCE, j_corr_ = 24 nA/cm^2^, E_pitt_ = −601 mV vs. SCE, c_b_ = 0.95 µF/cm^2^ 168 h immersion in 3 wt.% NaCl: E_corr_ = −735 mV vs. SCE, j_corr_ = 132 nA/cm^2^, E_pitt_ = −598 mV vs. SCE, c_b_ = 3.87 µF/cm^2^		RBS and GDOES confirmed the presence of Mo species: up to 0.15 at.% of Mo (RBS).	
AA 2024 T3	(40 g/L 1 H_2_SO_4_ (0.47 M) + 80 g/L C_4_H_6_O_6_ (0.53 M)	14 V, 37 °C, 20 min	EIS measurements were conducted in naturally aerated 0.5 M NaCl and acidified 0.1 M NaCl (pH = 4.0); XPS, RBS, and STEM-HAADF confirmed incorporation of the Mo species, up to 0.6 at% of Mo by RBS.			[86]
	(40 g/L 1 H_2_SO_4_ (0.47 M) + 80 g/L C_4_H_6_O_6_ (0.53 M) + 0.1 M Na_2_MoO_4_					
AA 5052	70 wt% H_4_P_2_O_7_	15 °C, 30 V, 0 min	E_corr_ = −0.729 V vs. Ag|AgCl, j_corr_ = 27.69 µA/cm^2^		Corrosion research was conducted in 3.5 wt.% NaCl, but at 70 C, to simulate the exploitation conditions; XPS and EDS confirmed the incorporation of Mo species into anodic alumina (up to 0.48 wt.%), as well as the incorporation of pyrophosphates (up to 2.49 wt.%).	[87]
		15 °C, 30 V, 20 min	E_corr_ = −0.715 V vs. Ag|AgCl, j_corr_ = 20.76 µA/cm^2^			
		15 °C, 30 V, 40 min	E_corr_ = −0.722 V vs. Ag|AgCl, j_corr_ = 21.47 µA/cm^2^			
		15 °C, 30 V, 60 min	E_corr_ = −0.729 V vs. Ag|AgCl, j_corr_ = 22.14 µA/cm^2^			
		15 °C, 30 V, 20 min	E_corr_ = −0.715 V vs. Ag|AgCl, j_corr_ = 20.76 µA/cm^2^			
		15 °C, 40 V, 20 min	E_corr_ = −0.702 V vs. Ag|AgCl, j_corr_ = 19.23 µA/cm^2^			
		15 °C, 50 V, 20 min	E_corr_ = −0.687 V vs. Ag|AgCl, j_corr_ = 17.66 µA/cm^2^			
		15 °C, 60 V, 20 min	E_corr_ = −0.668 V vs. Ag|AgCl, j_corr_ = 14.32 µA/cm^2^			
		15 °C, 30 V, 20 min	E_corr_ = −0.715 V vs. Ag|AgCl, j_corr_ = 20.76 µA/cm^2^ CPE_b_ = 1.36 µF/cm^2^, R_b_ = 3165 Ω·cm^2^		Barrier layer thickening with Mo amount increase; BL = 6.5 nm.	
	70 wt% H_4_P_2_O_7_ + 0.01 M Na_2_MoO_4_	15 °C, 30 V, 20 min	E_corr_ = −0.642 V vs. Ag|AgCl, j_corr_ = 17.55 µA/cm^2^, CPE_b_ = 1.17 µF/cm^2^, R_b_ = 3590 Ω·cm^2^		BL = 7.6 nm (0.13 wt.% of Mo; EDS)	
	70 wt% H_4_P_2_O_7_ + 0.1 M Na_2_MoO_4_	15 °C, 30 V, 30 min	E_corr_ = −0.528 V vs. Ag|AgCl, j_corr_ = 10.1 µA/cm^2^ CPE_b_ = 0.68 µF/cm^2^, R_b_ = 4548 Ω·cm^2^		BL = 13.0 nm (0.33 wt.% of Mo; EDS)	
	70 wt% H_4_P_2_O_7_ + 0.5 M Na_2_MoO_4_	15 °C, 30 V, 40 min	E_corr_ = −0.264 V vs. Ag|AgCl, j_corr_ = 5.67 µA/cm^2^ CPE_b_ = 0.58 µF/cm^2^, R_b_ = 6252 Ω·cm^2^		BL = 15.3 nm (0.48 wt.% of Mo; EDS); 0 h immersion in 3.5 wt.% NaCl, 70 °C	
			E_corr_ = −0.345 V vs. Ag|AgCl, j_corr_ = 8.86 µA/cm^2^ CPE_b_ = 1.54 µF/cm^2^, R_b_ = 5689 Ω·cm^2^		24 h immersion in 3.5 wt.% NaCl, 70 °C	
			E_corr_ = −0.428 V vs. Ag|AgCl, j_corr_ = 12.23 µA/cm^2^ CPE_b_ = 2.68 µF/cm^2^, R_b_ = 4985 Ω·cm^2^		72 h immersion in 3.5 wt.% NaCl, 70 °C	
			E_corr_ = −0.462 V vs. Ag|AgCl, j_corr_ = 13.29 µA/cm^2^ CPE_b_ = 2.93 µF/cm^2^, R_b_ = 4608 Ω·cm^2^		120 h immersion in 3.5 wt.% NaCl, 70 °C	
			E_corr_ = −0.488 V vs. Ag|AgCl, j_corr_ = 13.84 µA/cm^2^ CPE_b_ = 3.24 µF/cm^2^, R_b_ = 4257 Ω·cm^2^		168 h immersion in 3.5 wt.% NaCl, 70 °C	

Lv et al. reported interesting research on AA 5052 anodizing in 70 wt.% pyrophosphoric acid with various amounts of sodium molybdates [87]. The authors reported the impact of the anodizing voltage (30–60 V) and duration of the process (up to 60 min) on corrosion potential, corrosion current density, and wetting contact angle. A tremendous improvement in the corrosion performance of the formed oxide was achieved for the optimized conditions: the corrosion potential grew from −0.715 V vs. Ag|AgCl (70 wt.% H_4_P_2_O_7_, 15 °C, 30 V, 20 min) to −0.264 V vs. Ag|AgCl (70 wt% H_4_P_2_O_7_ + 0.5 M Na_2_MoO_4_, 15 °C, 30 V, 40 min). Simultaneously, the corrosion current density dropped from 20.76 µA/cm^2^ to 5.67 µA/cm^2^ (Table 4). Furthermore, the EIS study allowed the authors to confirm a significant improvement in the performance and thickness of the barrier layer, acknowledged by the application of sodium molybdate. The barrier layer capacitance dropped from 1.36 to 0.58 µF/cm^2^, which was accompanied by the barrier layer thickening from 6.5 nm (reference sample) to 15.3 nm (optimized samples with 0.5 M Na_2_MoO_4_). Also, the resistance of the barrier layer grew from 3165 to 6252 Ω·cm^2^. The anodized AA 5052 alloy finds application in distillation/desalinization water plants; thus, it is exploited in harsh environments. Therefore, the authors also performed a coating degradation study in 3.5 wt.% NaCl at 70 °C. 

The study reveals slow but gradual degradation of the coating with time, up to 168 h of immersion. For the optimized anodizing conditions, it was shown that corrosion potential drops from −0.264 V vs. Ag|AgCl to −0.488 V vs. Ag|AgCl. The corrosion current density grew from 5.67 to 13.84 µA/cm^2^. The EIS parameters for the barrier layer also worsened: CPE_b_ grew from 0.58 to 3.24 µF/cm^2^, while *R_b_* dropped from 6252 to 4257 Ω·cm^2^. Nevertheless, the corrosion data acquired for optimized conditions after 168 h of immersion in harsh environments are still better than for coatings anodized even with Na_2_MoO_4_, but for not optimized conditions, and lower inhibitor concentration. It is worth mentioning that these results are still outstanding when compared to the performance of coatings without molybdates.

## 5. Summary

Anodization of aluminum alloys is an effective corrosion protection method; however, applied Cr(VI)-based approaches were found to be toxic and have to be replaced with environmentally friendly methods. Up to now, various methods have been developed, and the application of electrolytes based on sulfuric acid with organic acids, like tartaric or citric acid, seem to be promising, but still not as good as chromic acid anodizing. Another step that can provide progress in Cr(VI)-free anodizing is the incorporation of various aluminum corrosion inhibitors into anodic alumina. It was shown that the anodic alumina has a duplex structure, in terms of chemical composition: the outer part forms a pure alumina skeleton, while the inner part, close to the pore center, is rich in the incorporated anions. Up to now, the most commonly applied inhibitors are cerium (IV) salts, potassium permanganate, and sodium molybdate. Potassium permanganate provides the best corrosion performance; however, it cannot be combined with tartaric or citric acid electrolytes because it oxidizes organic acids. A milder oxidizer that provides much better corrosion performance is sodium molybdate, when combined with sulfuric acid and organic acids. Therefore, the promising aim of future research will rather rely on a combination of organic acids, like tartaric or citric acid, in sulfuric acid baths containing mild inorganic oxidizers like molybdates.
